# Adaptation and constraint shape the evolution of growth patterns in passerine birds across the globe

**DOI:** 10.1186/s12983-020-00377-7

**Published:** 2020-09-30

**Authors:** Vladimír Remeš, Beata Matysioková, Jakub Vrána

**Affiliations:** 1 Department of Zoology and Laboratory of Ornithology, Faculty of Science, Palacky University, 17. listopadu 50, 77146 Olomouc, Czech Republic; 2 Department of Ecology, Faculty of Science, Charles University, Viničná 7, 12800 Praha, Czech Republic

**Keywords:** Birds, Development, Growth rate, Latitude, Life history, Nest predation

## Abstract

**Background:**

Growth trajectories should be adapted to selective factors of each species’ environment. However, major shaping forces of growth and development are unclear, especially when studying several traits at once. Birds provide an ideal opportunity to analyze growth patterns across species due to there being enough available data. We tested the relative importance of nest predation risk, the number of care-givers, nest height, foraging substrate, clutch size, and latitude on growth patterns of passerine birds (Passeriformes) using phylogenetic comparative methods. Specifically, we studied the evolution of fledging time, average and peak growth rates, and relative development at fledging of body mass and tarsus, wing, and tail length.

**Results:**

Using a comprehensive literature search and data quality control, we obtained data on growth in 231 species based on 295 populations. Species with long development in the nest grew slowly and had well-developed traits at fledging. Species breeding under high nest predation risk, building their nests close to the ground, and those living in northern temperate regions fledged early and grew fast, sometimes fledging with less developed body mass and traits critical for locomotion (tarsus, wing, and tail). On the other hand, the number of caring adults, clutch size, and species’ foraging substrate had very limited predictive value for growth patterns across passerine species.

**Conclusions:**

Shortening of the nestling period was a primary means of accelerating development (in relation to nest predation, nest height, and latitude), sometimes supplemented by higher peak growth rates of body mass, tarsus, and wing (especially in relation to latitude). Overall growth patterns of passerines were adaptively tuned to nest predation risk and nest height, with northern temperate species having especially short nestling periods and fast growth rates of body mass, tarsus, and wing.

## Background

Size and shape of organisms are critical for their survival and reproduction [1]. Adaptations in adult morphology have been documented in a broad range of taxa and are classical examples of adaptive design in evolutionary biology [2], for example in terms of movement or feeding [3–5]. However, adult morphology is mostly a result of developmental trajectories during ontogeny [6]. Consequently, it is critical to understand the origin of adult form for at least two reasons. First, growth and developmental patterns might carry-over to adulthood affecting adult structure and performance, for example in frogs [7] and birds [8]. Second, patterns of growth and development might be themselves adaptive in a given set of environmental conditions [9], and adaptations in terms of plastic responses within species have been investigated extensively [10]. Yet, ontogenetic growth patterns of multiple traits have rarely been examined in relation to species ecology and life history across a broad range of species, with few exceptions [11].

Birds are an ideal group to investigate ontogenetic patterns and differential growth of traits [12, 13]. First, we know that growth rate of body mass is adaptive across species: young birds grow fast and leave the nest early in species under strong nest predation pressure to avoid time-dependent mortality [14–16]. Moreover, growth rates seem to be adaptively tuned to a theoretically predicted trade-off between survival in the nest and post-fledging survival [17]. Accordingly, species under strong nest predation pressure fledge early, but at a cost of lower post-fledging survival [8, 18]. However, all these observations have been based solely on the growth rate of body mass. Yet, differential growth of locomotor traits (tarsus, wing, tail) might be tightly integrated into overall species life history. First, growth rates of wings and tarsus increased with nest predation risk in 12 species of passerines in a high elevation site in Arizona [11]. This might have clear benefits for post-fledging survival, as juvenile flight ability increased with increasing development of wings at fledging in 13 species of passerines in Illinois [19]. Second, wing growth rate might be a key component of life history explaining small tropical clutches. More specifically, tropical birds are supposed to invest in few, high-quality offspring that fledge with long wings and thus are better able to escape high predator pressure upon fledging [16, 20, 21]. Thus, investigation of differential growth of locomotor traits and their ecological and behavioral correlates is very important for understanding their diversification and overall integration of avian life histories.

Nest predation is a strong environmental factor selecting for fast growth of body mass and wings as an adaptation to escape time-dependent juvenile mortality in the nest [11, 14–16, 21]. However, at least three further factors might affect the evolution of growth rates of locomotor traits in birds. First, energy available per capita might constrain growth rate [16], especially given that fast growing young have higher intensity of metabolism and thus energy consumption [22]. It is thus possible that young in species with many offspring in the nest obtain less food per capita [15] and might grow slowly [23, 24]. On the other hand, food delivery might depend on the number of feeding adults. For example, nests/species with only one caring adult can have low feeding rates [15, 25], while those with many caring adults can have high feeding rates [26–29]. Consequently, slower growth might be expected in species with female-only parental care, while faster growth might be expected in cooperative breeders with helpers.

Second, nest height and foraging substrate might affect relative growth of locomotor traits by constraining or facilitating certain evolutionary options. It might pay ground nesters to grow fast, because they can move around the nest early and with relatively undeveloped locomotor capabilities without elevating their post-fledging mortality [30]. Consequently, they can fledge early, while that could be fatal for canopy nesters (i.e., young falling on the ground risking injuries). Ground nesters can in this way escape the risk of whole-brood depredation while being able to feed the family on the territory [21]. This argument assumes that these effects are additive to nest predation risk, because ground nesting makes available (i.e. “facilitates”) growth patterns that are unavailable to species nesting higher above the ground. In terms of foraging substrate, species foraging on vegetation and especially in the air might need relatively slow growth and long development so that their tarsi and wings are appropriately developed and more functional for demanding foraging strategies [31–33], while ground foragers are not constrained in this way.

Third, latitude affects avian life histories: tropical species of birds have low energy expenditure [34] and invest in few, high-quality young [16]. They also have lower peak growth rates of body mass [15, 23, 35] but better developed wings at fledging [16] which brings the benefit of better flight performance and post-fledging survival [19, 21]. Moreover, tropical species often differ from species living in higher latitudes in various life-history and behavioral characteristics; for example, they have higher adult survival, longer post-fledging care, or their activity around the nest is more sensitive to depredation risk [8, 36, 37]. Nevertheless, a study of growth patterns including growth of both mass and all locomotor traits across latitudes has never been conducted.

To advance our understanding of ecological and evolutionary factors shaping growth of locomotor traits in birds, we conducted a global comparative analysis of nestling growth in songbirds (Passeriformes). We studied growth patterns in 231 species based on 295 populations across six continents spanning the full latitudinal gradient from the tropics to arctic regions. Specifically, we studied the evolution of fledging time, average and peak growth rates, and relative development at fledging of body mass, and tarsus, wing, and tail length. We studied growth patterns in relation to nest predation rates, the number of care-givers, clutch size, nest height, foraging substrate, and latitude (our predictions are summarized in Table 1).

**Table 1:** Summary of tested hypotheses on the evolution of growth patterns in passerine birds

Factor	Reasoning	Predictions for growth rates	Predictions for trait development at fledging and fledging age
**Time-dependent mortality**			
Nest predation	High risk of nest depredation selects for fast growth and early fledging.	Faster growth rate under high nest predation. Growth rate of wings might be especially prioritized to enable escaping from predators.	Early fledging, potentially with less developed locomotor traits, under high nest predation.
**Energy**			
No of caregivers	Higher energy availability enables fast growth and better trait development.	Growth rate faster with more care-givers (Cooperative breeding > pairs > female-only care).	Early fledging and/or better development of locomotor traits with more care-givers.
Clutch size	Energy requirements increase with both chick number and growth rate.	Slower growth in large broods due to energetic constraint.	Delayed fledging or less developed locomotor traits in large broods.
**Constraint/Opportunity**			
Nest height	Ground nesting enables early fledging and thus escaping whole-brood depredation (for a given nest predation rate).	Fast growth in ground-nesters; slower growth in species nesting higher up.	Less developed locomotor traits in ground-nesters; better trait development and delayed fledging in species nesting higher up.
Foraging substrate	Ground foraging enables early fledging and thus exploitation of resources by parents; vegetation and air foraging requires high-quality locomotor traits, esp. wings.	Fast growth in ground-foragers; slower growth in species foraging on vegetation and especially in the air (wings).	Less developed locomotor traits in ground-foragers; better trait development and delayed fledging in species foraging on vegetation and especially in the air (wings).
**Geography**			
Latitude	Tropical species have overall slow life history and invest into fewer, higher-quality young.	Slow growth in tropical species.	Better developed locomotor traits and delayed fledging in tropical species.

## Methods

### Data collection

We collected data for this study from the literature during the last 15 years of our work on avian growth [14, 17, 38, 39], incubation [37, 40, 41], and nest predation [42, 43]. Our data collection protocols include systematic searches of handbooks and journals, database searches, and searching reference lists of relevant articles.

From the original studies, we extracted information on the location of the study. We used this information to obtain the latitude and longitude where the study was conducted using Google Earth. We then extracted primary data on the growth of body mass, wing length, tarsus length, and tail length when the young were in the nest, i.e. up to fledging. This data came from tables and figures in the original articles. If possible, we also noted the number of nests and/or nestlings weighed and measured. We also extracted fledging age, body mass at hatching, and body mass, wing length, tarsus length, and tail length at the day of fledging. Nestling mass must have been weighed within 10% of fledging age to be included as body mass at hatching. For example, in species staying for 20 days in the nest, the hatching mass must have been weighed between days 0 and 2. We thus obtained data on growth in 456 populations of 336 passerine species. Average number of populations per species was 1.4 (median = 1, range = 1–7). Of course, not all source studies provided data on all traits and thus sample sizes differed across analyses. The numbers of studies providing data on particular characteristics can be obtained from our data set (Additional File 3), while sample sizes for particular analyses can be obtained from supplementary tables (Additional File 1).

We then used ornithological handbooks (see Table S1 in Additional File 1) to find information on relevant predictors and covariates at the species level. These included adult values of body mass, wing length, tarsus length, and tail length. If these data were given separately for males and females, we used their arithmetic average, because the sex of nestlings is almost never known, which precludes sex-specific analyses of growth. We further obtained data on clutch size (number of eggs in a complete clutch), nest height (in meters), the number of care givers during the nestling period (female-only, breeding pair, cooperation of more than two individuals), and the substrate of food collection (ground, vegetation, and air). For justification of these predictors and covariates, see Introduction and for associated predictions, see Table 1. Lastly, we searched primary literature for data on nest predation rates in our sample of species. We succeeded in finding nest predation information as the percentage of depredated nests (with the minimum sample size of 10 nests) in 187 species. We then converted these percentages to daily nest predation rates (i.e., probability of nest depredation per 1 day [43]). Please note that nest predation data came from different populations than growth data. This might have introduced noise into our analyses.

### Data processing

We calculated three characteristics of growth rate in nestling passerines at the population level. First, we calculated the ratio of the population-specific trait value at fledging with adult values. If available, we used population-specific adult values, otherwise we used species-specific adult values. We call these ratios relative fledging mass, relative fledging wing length, relative fledging tarsus length, and relative fledging tail length, respectively. They are measures of the relative development of the young when leaving the nest. Relative fledging mass and relative fledging wing length are good predictors of post-fledging flight performance [19] and survival of fledglings in passerines [8, 21].

Second, we estimated two indices of growth rate in the nest. We estimated peak growth rates using the parameter *K* of a sigmoid growth function, and we did this for body mass, wing length, and tarsus length (tail length was not fitted due to problems with convergence in too many populations). We estimated peak growth rate also when body mass of nestlings was cut at 70% of adult mass, which might improve estimates of growth rates [14]. We could not use the 70% cut-off for other traits due to lack of convergence in most cases. The parameter *K* is independent of overall body mass and thus is a convenient, scale-free measure of peak growth rate. There are several sigmoid functions used to estimate growth rates in animals (Fig. S1 in Additional File 2), but the logistic function is overwhelmingly used in studies of growth in small birds [14–16, 23]. It is a three-parameter model where the nonlinear fitting procedure estimates peak growth rate (*K*), asymptote of the sigmoid curve (*A*), and the age of inflection (*t*_*i*_, the point in time where the growth trajectory changes from accelerating to decelerating [14]).

However, the main caveat of relying on a single three-parameter model is that the model may not be flexible enough to return accurate parameter values [44]. We thus also fitted the four-parameter Richards growth function that besides the three above-mentioned parameters estimates also the shape parameter *d*, which flexibly places the inflection point on the trait (i.e., vertical) axis between 0 and the estimated asymptotic value *A* [45]. On the contrary, the logistic function fixes the position of *d* at 50% of *A* [14]. We thus used both the traditionally used logistic function and more flexible Richards function as recommended by a recent modelling study of passerine growth [46]. We used Unified versions of both sigmoid functions (U-logistic and U-Richards), which ensures comparability of *K* estimates across different growth functions [46, 47]. One of the potential problems identified with fitting sigmoid growth functions to growth data can be poor estimation of the upper asymptote *A*, which might bias estimates of *K* [14, 48]. We thus present evidence that our estimates of *A* were close to adult trait values across our sample of species (mean correlation coefficient was *r* = 0.86, *n* = 8 values; Figs. S2 and S3 in Additional File 2). We also show that fitting of growth curves with the asymptote being estimated vs. fixed at adult value give highly positively correlated estimates of peak growth rates (mean correlation coefficient was *r* = 0.74, *n* = 6 values; Table S2 in Additional File 1).

Further, we calculated average growth rate of body mass, wing length, tarsus length, and tail length. We calculated it as log(trait at fledging)/development time. It is sometimes called “relative growth rate” (in g g^− 1^ day^− 1^) and technically it is the derivative of log(trait) over time [49, 50]. Its calculation brings two problems. First, it assumes exponential growth that is rare in animals [51]. Passerine growth in the nest is typically sigmoidal and decelerating on the log-linear scale (Fig. S4 in Additional File 2), and thus instantaneous relative growth rate also decreases with time. Second, log(trait at fledging)/time assumes the same starting trait value at hatching. A better estimate would be log(trait at fledging/trait at hatching)/time, as was argued also for seed size in plants: log(plant size/seed size)/time [52, 53]. Nevertheless, estimates of hatchling size are rare due to difficulties of measuring tiny hatchlings, with the exception of body mass. We thus show that i) estimates of average mass growth rates obtained using the two methods are highly positively correlated (*r* = 0.81; Fig. S5 in Additional File 2), and ii) results obtained with estimates of average growth rate calculated without hatchling mass are similar to those obtained with hatchling mass (Table S3 in Additional File 1). Despite all the caveats mentioned above, we used average growth rates in addition to peak growth rates, because the latter do not express overall growth achieved in the nest and average growth rates can thus bring additional insights into the evolution of growth patterns (see below). It is also important to realize that average growth rates are the same irrespective of a particular growth trajectory in the nest, and thus their estimation can be more robust than estimation of peak growth rates, the latter coming with their own errors and biases [14, 48].

### Statistical analyses

We used phylogeny-based comparative methods to test our hypotheses. We downloaded 500 phylogenetic trees for our species from a publicly available archive at birdtree.org using Hackett constraint, all species, and version 2 (V2) of the archive [54]. We calculated one Bayesian maximum credibility tree using TreeAnnotator [55] and used this tree in our comparative analyses. We analyzed our data at the species level using the mean values of response variables calculated for each species across populations available for that particular species. To obtain mean values across populations, we first calculated point estimates of a given variable (e.g. wing growth rate) for individual populations and then took arithmetic means of these point estimates across all available populations for a given species. We used phylogenetic generalized least squares, PGLS [56], to fit our multiple regression models on species means in the *ape* [57] and *caper* packages [58] for R 3.5.1 software. We checked that residuals from these models were normally distributed and homoscedastic and that there were no non-linearities [59].

We selected only populations where the sample size was known and at least five nestlings were measured. We then checked that this procedure increased the quality and reliability of our data by calculating within-species repeatability of population estimates as the intraclass correlation coefficient using the *ICC* package for R software [60]. Indeed, repeatability of our dependent variables increased on average from 0.36 to 0.57 in peak growth rate *K* (Fig. S6 in Additional File 2) and from 0.87 to 0.91 in fledging values of tarsus, wing and tail length, body mass, and brood age (Fig. S7 in Additional File 2). Although repeatability of population-level estimates of our dependent variables was moderate to high, remaining within-species variation might still affect parameter estimates. Moreover, phylogenetic uncertainty could also affect parameter estimates. We dealt with both these problems by fitting a subset of our models also on population-level data across several phylogenies using phylogenetic mixed models implemented in the *MCMCglmm* package [61] as in our previous work [37]. However, results were similar to those obtained using species-level PGLS and we thus report only PGLS results and provide all data needed to replicate our analyses or use other fitting methods.

We modelled three types of response variables: i) fledgling traits, ii) fledging age, and iii) growth rates. Fledgling trait is the value of a given trait (body mass, tarsus, wing, and tail length) at the time of fledging, and we used either absolute trait value or relative trait value (see above). Fledging time is the age of nestlings at the time of fledging (in days). Growth rates are either average growth rates or peak growth rates estimated by parameter *K* of a sigmoid growth function (either Logistic or Richards). Peak growth rates were modelled either as “absolute” peak growth rate, where we put the adult value of a given trait among predictors, or as “relative” peak growth rate, where we put growth rate of body mass among predictors. Thus, in the first case, it was a classic allometric adjustment, while in the second case we modelled trait growth rate (tarsus and wing length) *relative* to body mass growth rate.

We scaled all continuous variables (subtracted mean and divided by one standard deviation) to obtain parameter estimates comparable across variables and models. However, parameter estimates for factors (the number of care givers and substrate of food collection) were still not comparable to other estimates [62]. Many variables were skewed and thus we used log10 or square root transformations to improve their distribution. As dependent variables, we used log10(fledging trait), and log10(fledging time), while growth rates remained untransformed. As predictor variables, we used log10(daily nest predation rate + 0.01), square root(nest height), square root(clutch size), and log10(adult trait), while other predictors remained untransformed. These transformations are also noted in tables reporting results in the Additional File 1.

Clutch size was strongly correlated with absolute latitude (*r* = 0.66, *n* = 453 at the population level; *r* = 0.69, *n* = 333 at the species level for all species; *r* = 0.68, *n* = 229 at the species level for species entering analyses; clutch size log10-transformed). To avoid collinearity of predictors, we fit two sets of models. One set with latitude without clutch size and the other with both latitude and clutch size included. When studying geographic effects of latitude, we used three strategies. First, we fitted absolute latitude as a predictor, because many life-history traits change systematically with increasing distance from the equator in birds [15, 16]. Second, we also fitted an interaction of absolute latitude with hemisphere (northern vs. southern) to find out whether the slope of latitudinal effect differed between the hemispheres. This interaction was, however, never statistically significant and thus we omitted it from all models. Third, tropical, southern temperate, and northern temperate birds (delimited by 23.5°N and 23.5°S) often differ in their life histories and behavior [8, 36, 37]. We thus also fitted latitudinal band (northern temperate, tropical, southern temperate) as a predictor in our models that excluded absolute latitude.

We obtained data on nestling growth and development from 456 populations of 336 species of passerines worldwide (dataset available in Additional File 3). However, in 84 populations the number of measured nestlings was not known, while in 77 populations it was lower than five. Thus, we ended up with 295 populations of 231 species where the number of measured nestlings was known and was at least five (Fig. S8 in Additional File 2). The most limiting predictor was nest predation rate, which was available for only 152 out of these 231 species and was not available in the remaining 79 species. Thus, our final sample size that was used in all analyses was 152 species, but it was usually lower due to lacking other variables (mainly growth rates and fledging values of individual traits). Scaling analyses that did not include nest predation were the only exception, because there we were able to use up to 230 species.

## Results

**Fig. 1:**
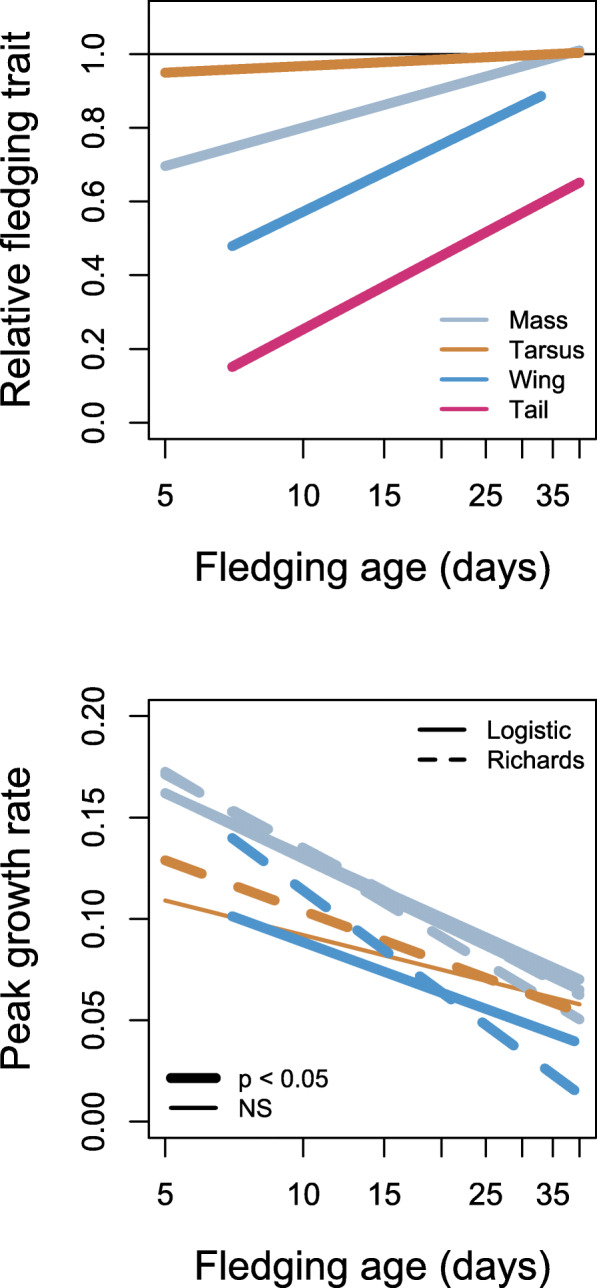
Relative development of traits at fledging (top) and peak growth rates (bottom) in relation to fledging age in passerines. In relative trait development, the value of 1 means that the trait was developed at 100% of adult value at fledging, and this is designated by a horizontal line. Growth rate is the peak growth rate (*K* parameter) from a sigmoid growth model (either three-parameter U-Logistic or four-parameter U-Richards model). There are two lines for mass growth (for both Logistic and Richards models); one is for complete nestling data, while the other is for nestling body mass truncated at 70% of adult mass. These relationships remained unchanged when growth rates and fledging age were adjusted for allometry (see Figs. S12–S17 in Additional File 2 and Tables S4 and S5 in Additional File 1)

Most species fledged with well-developed tarsus (mean = 97.5% of adult value, range = 67.1–110.1%, *n* = 170 species) but comparatively underdeveloped tails (mean = 37.1%, range = 3.7–100%, *n* = 72). Relative development of wings (mean = 65.7%, range = 26.6–100%, *n* = 129) and body mass (mean = 84.8%, range = 41.0–121.7%, *n* = 231) were intermediate (Fig. S9 in Additional File 2). Large species had low relative fledging mass, while relative development of other traits did not scale with adult values (Figs. S10 and S11 in Additional File 2). Species with long nestling periods had well-developed traits at fledging (Fig. 1 and S12 in Additional File 2). This relationship was not driven by large species having both long nestling periods and well-developed traits at fledging, because it was the same, or even more apparent, when fledging age was corrected for adult body mass (Fig. S13 in Additional File 2, Table S4 in Additional File 1). At the same time, species with long nestling periods had slow peak growth rates of all traits (Figs. 1, S14 and S15 in Additional File 2) and this was also true for peak growth rates and fledging age adjusted for allometric relationships with adult trait values (Figs. S16 and S17 in Additional File 2; Table S5 in Additional File 1). These correlations combined into fast growing species having less developed traits at fledging, with the exception of tarsus length (Figs. S18 and S19 in Additional File 2, Table S6 in Additional File 1).

### Nest predation

**Fig. 2:**
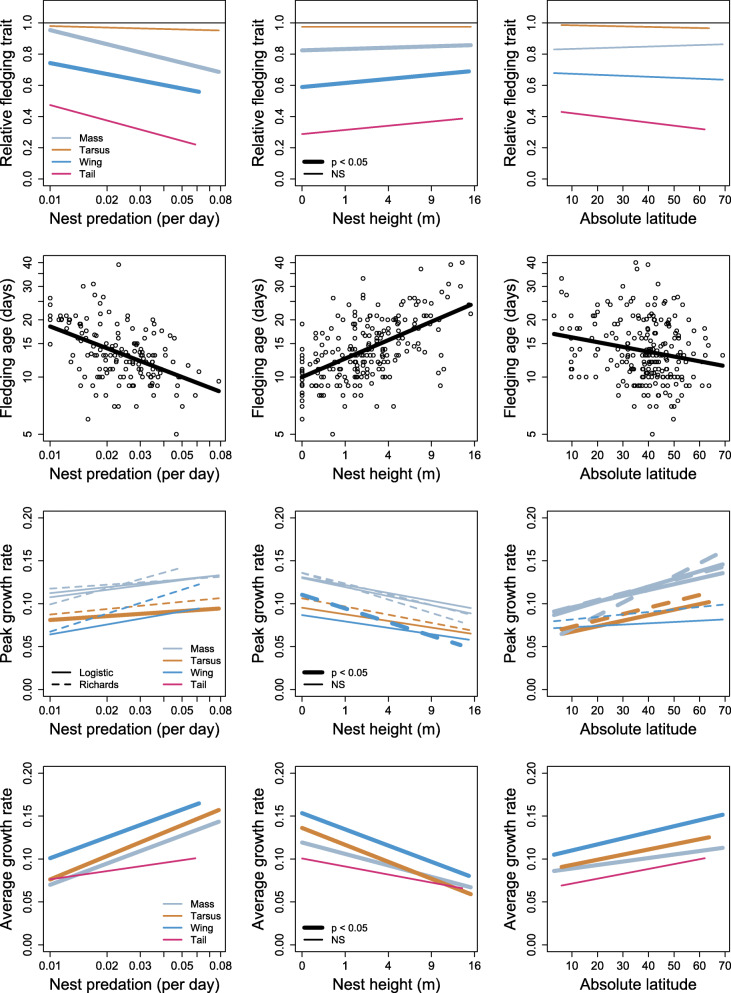
Relationships between traits characterizing growth patterns of passerines (relative size and mass at fledging, fledging age, and growth rates) and most important predictors (nest predation risk, nest height, and absolute geographic latitude). For statistics, see Tables S7–S12 in Additional File 1

**Fig. 3:**
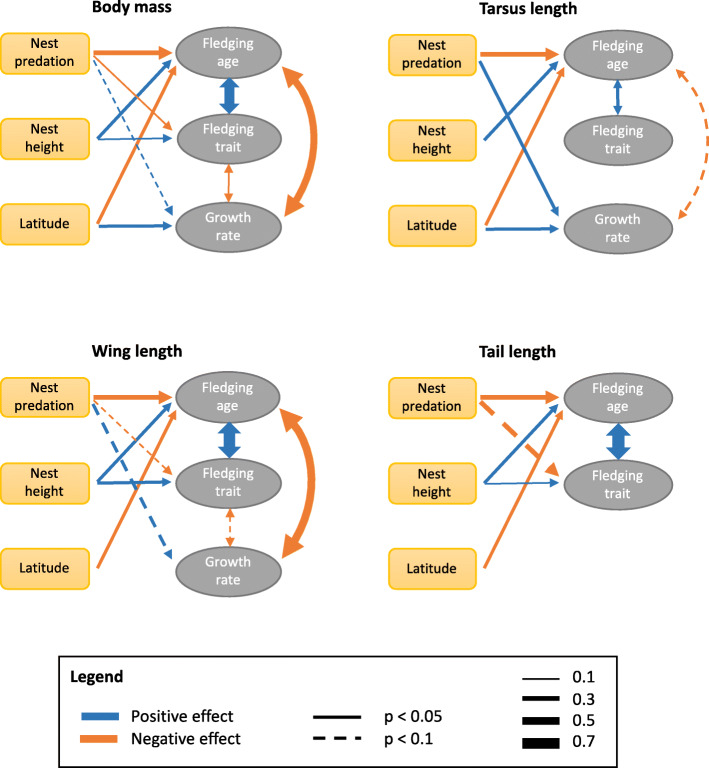
Summary of relationships among predictors (yellow rectangles) and dependent variables (grey ovals). Models were fit using phylogenetic generalized least squares regressions. Effect size is a standardized regression coefficient and is depicted by path width. We did not use phylogenetic path analyses, because that would lead to substantially reduced sample sizes. “Growth rate” is peak growth rate (U-Logistic curve) and it was not estimated for tail length (see Methods). “Latitude” is a geographic latitude, increasing away from the equator. For statistics, see Tables S7–S12 in Additional File 1

Species with high nest predation rates fledged significantly earlier and at lower body mass with shorter wings and tails than species with low nest predation rates (Figs. 2 and 3, Tables S7–S9 in Additional File 1). Average growth rates of mass, tarsus, and wings increased significantly with increasing nest predation rate (mean size of standardized regression coefficients = 0.29 [range 0.26 to 0.32, *n* = 3 estimates]; Figs. 2 and 3, Table S10 in Additional File 1). Effects of nest predation on peak growth rates were weaker (mean effect size = 0.15 for absolute peak growth [range 0.02 to 0.21, *n* = 8 estimates of which only one was statistically significant], and 0.14 for relative peak growth [range 0.06 to 0.24, *n* = 8 estimates of which only one was statistically significant]; Figs. 2 and 3, Tables S11 and S12 in Additional File 1).

### Number of care-givers and clutch size

The number of care-givers (female-only, pair, and cooperative parental care) never had a statistically significant effect on any component of growth (Tables S7–S9 in Additional File 1). Species with larger clutch sizes fledged with significantly longer wings (Tables S8 and S9 in Additional File 1) and had significantly slower average growth rate of wings (Table S10 in Additional File 1). Both these effects were apparent only when latitude was treated as the latitudinal band (northern temperate, tropical, and southern temperate), not when in was treated as a continuous variable. This suggests a potentially confounding effect of collinearity between clutch size and latitude.

### Nest height and foraging substrate

Species nesting higher above the ground fledged significantly later and at higher mass with longer wings than species nesting closer to the ground (Figs. 2 and 3, Tables S7–S9 in Additional File 1). Average growth rates of mass, tarsus, and wings all decreased significantly with increasing nest height above the ground (mean size of standardized regression coefficients = − 0.23 [range − 0.19 to − 0.26, *n* = 3 estimates]; Figs. 2 and 3, Table S10 in Additional File 1). Effects of nest height on peak growth rates were on average weaker and more variable (mean effect size = − 0.12 for absolute peak growth [range − 0.02 to − 0.27, *n* = 8 estimates of which only one was statistically significant], and − 0.22 for relative peak growth [range − 0.05 to − 0.40, n = 8 estimate of which only two were statistically significant]; Figs. 2 and 3, Tables S11 and S12 in Additional File 1). However, an overall trend for slower peak growth rate of wings in species nesting higher above ground was apparent, as in addition to the three relationships being statistically significant, further four were close to being statistically significant (with *p* < 0.1; Tables S11 and S12 in Additional File 1). Foraging substrate was a weak predictor of growth patterns. The only significant relationships were short relative tails at fledging in ground-foraging species and slow peak relative growth of wings (only Logistic, not Richards) in aerial-feeding species (Tables S7–S9 in Additional File 1).

### Latitude

**Fig. 4:**
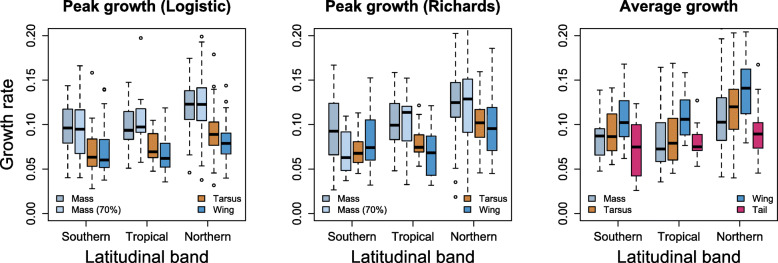
Growth rates of passerine nestlings in relation to latitude, expressed as geographic bands delimited by the Tropic of Capricorn (23.5°S) and Tropic of Cancer (23.5°N). “Southern” means south of 23.5°S, “Northern” means north of 23.5°N, while “Tropical” means between the two Tropics. For statistics, see Tables S10–S12 in Additional File 1

Species breeding further from the equator fledged earlier than species breeding close to the equator (Fig. 2, Table S7 in Additional File 1). Latitude did not clearly predict trait size at fledging (Fig. 2; Tables S8 and S9 in Additional File 1). On the contrary, its predictive value for growth rates was obvious. Species breeding further from the equator grew significantly faster than species breeding close to the equator (Figs. 2 and 3). The only exceptions where the relationships were not statistically significant were the average growth of the tail and peak growth of the wing, both absolute and relative (Fig. 2, Tables S10–S12 in Additional File 1). When split into latitudinal bands, virtually all latitudinal effects that were statistically significant (see above) were driven by northern temperate species. They fledged earlier and grew faster than tropical species, while southern temperate species did not differ from tropical species (Fig. 4, Tables S7, S10–S12 in Additional File 1).

## Discussion

**Fig. 5:**
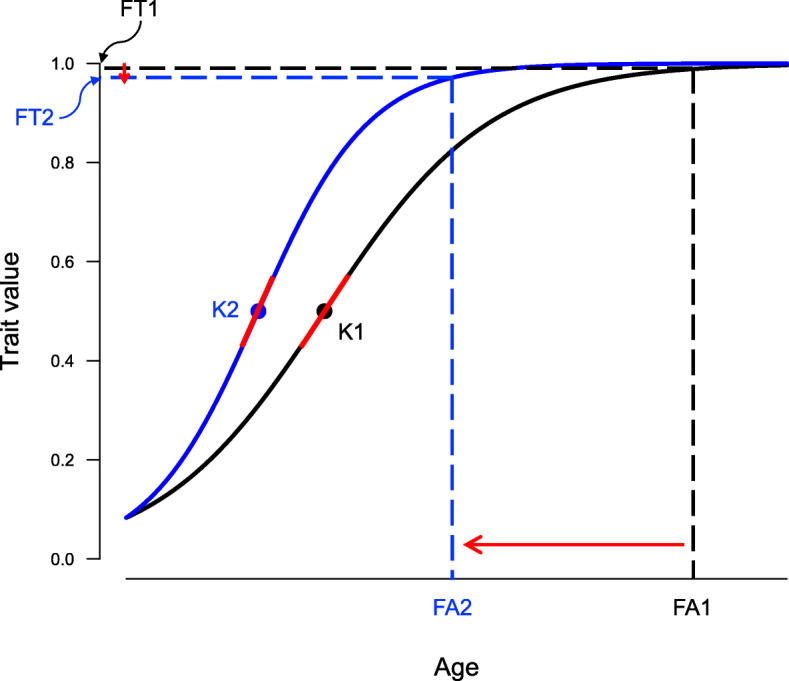
Evolutionary responses of growth in passerines to ecological factors. The strongest response to high nest predation risk, building nests close to ground, and living in northern temperate latitudes is shortening of fledging age (red arrow from FA1 to FA2). Due to the sigmoid shape of typical growth trajectory, this brings only a small decrease in relative fledging traits (from FT1 to FT2). However, due to a strong response in fledging age, it is tied to strong increase in average growth rate (where fledging age is in the denominator of the formula for its calculation). Finally, peak growth (slope of a tangent of the growth curve at the inflection point, depicted here as a red line) is sometimes also higher (increase from K1 to K2), especially for the latitudinal effect (see Figs. 2 and 3). Also note that longer fledging age (FA1 > FA2) is correlated with slower peak growth (K1 < K2) and larger fledging traits (FT1 > FT2, see Fig. 1)

We tested to what extent nest predation, number of care-givers, clutch size, foraging substrate, nest height, and latitude predicted growth patterns in passerines worldwide (Table 1). We showed that species breeding under high nest predation risk, building their nests close to the ground, and living in northern temperate regions fledged early and grew fast, sometimes fledging with less developed body mass and locomotor traits (Fig. 5). On the other hand, the number of caring adults, clutch size, and species’ foraging substrate had very limited predictive value for growth patterns across passerine species.

### Nest predation

Nest predation risk has been identified previously as a key agent selecting for early fledging and fast growth of body mass [14–16, 63] and wings in passerines [11, 21]. We confirmed these findings and showed that tarsus growth was accelerated under strong nest predation risk as well. At the same time, we found the strongest statistical effect in shortening fledging age. Since average growth is calculated as body mass increase divided by the fledging age, this short fledging age translated into faster average growth (meaning that the nestling reached the same body mass in shorter time). On the contrary, effects on peak growth rate were weaker and mixed. In particular, we found only a very weak increase of the relative growth rate of wings in relation to high nest predation rate, in contrast to previous findings [11]. This is surprising given long wings at fledging bring better nestling mobility [64] and decrease fledgling depredation rate [21, 30]. Short fledging time led to less developed traits at fledging, which can carry over to low post-fledging survival, driven mainly by relatively short wings at fledging [8, 18, 19, 21]. All these findings are consistent with a model for the evolution of fledging time in birds published by Roff et al. [17], although its direct parametrization is currently prevented by the lack of appropriate quantitative data.

### Number of care-givers and clutch size

If energy per capita was constraining growth rate, we would expect fast growth with many care-givers and small clutches. However, we found virtually no effects of the number of care-givers on growth patterns. Moreover, the effects of clutch size were quite conflicting from the energetic point of view. While large clutches correlated with slower wing growth, they also correlated with longer wings at fledging. The first finding was expected, as fast growing young have higher intensity of metabolism and thus probably higher rate of energy consumption [22], which might limit brood size. On the other hand, the energetics of growth and its effects on the evolution of growth patterns is far from clear. While Martin [16] showed that higher per capita feeding rates were correlated with relatively long wings at fledging across species, Martin et al. [15] found that species with higher nest predation rates grew faster than species with lower nest predation rates even when nestlings were getting less food per capita from their parents. Thus, either higher feeding per capita has variable effects on growth rates, or else higher number of care givers in relation to brood size does not always translate into higher per capita feeding rates or food load [65]. The reason for the latter might be strategic reduction of parental effort in parents with more helpers in terms of egg size [66] or feeding rate [27]. In line with our evidence, a study of shorebirds and gulls also found no effect of the number of caring parents (one vs. two) on chick growth rates [67]. Similarly, effects of brood size on growth rates were also mixed [14, 15, 23], potentially reflecting a conflict in larger broods between the energetic constraint selecting for slow growth and sibling competition selecting for fast growth [24, 38]. Unfortunately, quantitative data relating feeding rates/food load to the number of care givers and brood size across bird species are lacking, thus preventing a more direct test of the energy hypothesis.

### Nest height and foraging substrate

Nest height was a surprisingly strong predictor of growth in passerines. Species nesting high above the ground had long nestling periods and fledged with relatively long wings. Long nestling periods also translated into slow average growth rates. There was a strong trend for slow peak growth rate of wings in species nesting high above the ground. All these results agree with the idea that nesting high up in the canopy selects for the development of well-functioning locomotor traits, especially wings [33], at fledging. Leaving the nest with not fully functional wings could be fatal to the young bird, either due to injuries or failure to come back to the nest. On the other hand, ground nesters might be able to fledge early, which prevents whole-brood depredation. For example, only 9% of grey-headed juncos (*Junco hyemalis*) lost the entire brood of fledglings to mortality, whereas 38% of entire broods of nestlings found by a predator were depredated in the nest [21]. In contrast to nest height, foraging substrate was a weak predictor of growth patterns. Parents are probably able to supply enough food to the fledglings no matter what their foraging substrate is, because species have probably evolved to forage efficiently in whatever foraging substrate they occupy. This might alleviate any selection on nestling growth.

### Latitude

Development was fast away from the equator, which included early fledging and fast growth rates of body mass and tarsus and wing length [35, 68]. This pattern was driven by northern temperate species differing from tropical species, while southern temperate species did not differ from tropical species. This echoed previous findings, where northern temperate species had faster growth than tropical species, with southern temperate species falling in between [15, 63]. Early fledging and fast growth probably cancelled out in the present study, with no net effect of latitude on trait development at fledging. This contrasts with previous findings of Martin [16], who found faster relative peak growth of wings and relatively longer wings at fledging in tropical species as compared to northern temperate species. However, this conflict is at least partly resolved by our lack of finding faster peak growth rates of wings (both absolute and relative) in northern temperate species. Wing length was the only trait in our study that did not have faster peak growth rates in northern temperate species. This at least partly reconciles our findings with those by Martin [16].

It is difficult to explain faster growth in northern temperate species, because two explanations often put forward, namely nest predation and adult mortality, likely fail. First, nest predation was controlled for statistically in this study and thus could not have indirectly caused the significant positive effect of northern latitudes on growth rates. Second, an extensive study of 90 passerine species across four continents showed that adult mortality rate probably did not explain variation in growth rates across latitudes [63]. An explanation worth considering might be season length, because species with short breeding seasons had fast growth in shorebirds and gulls [67]. Thus, a pressure to finish development early and/or allow for re-nesting after failure within a short breeding season might be a strong selection on fast development. Indeed, within-species studies demonstrated that northern populations have shorter incubation and nestling periods, faster growth and higher brood mass for the same age than southern populations [64, 69–72]. Additionally, faster growth in northern temperate species can also be explained by the increased length of daytime during breeding period. Days are longer and nights shorter in arctic and temperate regions and breeding birds have more time available to feed nestlings [64, 70, 73, 74].

## Conclusions

Shortening of the nestling period was a primary means of adaptation to accelerate development, sometimes supplemented by higher peak growth rates, especially in relation to latitude. Overall growth patterns of passerines were finely tuned to nest predation risk and nest height. Moreover, northern temperate species had especially fast growth and development.

## Supplementary information

**Additional file 1: Table S1.** References used to obtain data. **Table S2.** Correlations between peak growth rates estimated with free vs. fixed asymptote. **Table S3.** Comparison of different estimates of average growth rate of body mass. **Table S4.** Relative traits at fledging in relation to adult body mass and fledging age. **Table S5.** Peak growth rates in relation to adult body mass and fledging age. **Table S6.** Trait values at fledging in relation to adult trait values and peak growth rates. **Table S7.** PGLS models of fledging age. **Table S8.** PGLS models of absolute trait values at fledging. **Table S9.** PGLS models of relative trait values at fledging. **Table S10.** PGLS models of average growth rates. **Table S11.** PGLS models of peak growth rates (“absolute”). **Table S12.** PGLS models of peak growth rates (“relative”).

**Additional file 2: Figure S1.** Sigmoid growth curves used to quantify peak growth rates. **Figure S2.** Asymptotes vs. adult trait values (Logistic). **Figure S3.** Asymptotes vs. adult trait values (Richards). **Figure S4.** Growth trajectories of 50 passerines. **Figure S5.** Average growth rates estimated using two different methods. **Figure S6.** Repeatability of peak growth rates across populations. **Figure S7.** Repeatability of fledging trait values across populations. **Figure S8.** Distribution of study populations across the world. **Figure S9.** Relative development of traits at fledging across species of passerines. **Figure S10.** Relative development of traits at fledging in relation to adult trait values in passerines. **Figure S11.** Fledging mass and fledging tarsus, wing, and tail length in relation to adult trait values in passerines. **Figure S12.** Relative development of traits at fledging in relation to fledging age in passerines. **Figure S13.** Relative development of traits at fledging in relation to residual fledging age in passerines. **Figure S14.** Peak growth rates (parameter *K* of the U-Logistic function) vs. fledging age in passerines. **Figure S15.** Peak growth rates (parameter *K* of the U-Richards function) vs. fledging age in passerines. **Figure S16.** Residual peak growth rates (parameter *K* of the U-Logistic function) vs. residual fledging age in passerines. **Figure S17.** Residual peak growth rates (parameter *K* of the U-Richards function) vs. residual fledging age in passerines. **Figure S18.** Relative development of traits at fledging in relation to residual peak growth rate (parameter *K* of the U-Logistic function). **Figure S19.** Relative development of traits at fledging in relation to residual peak growth rate (parameter *K* of the U-Richards function).

**Additional file 3.** Dataset used in all analyses for Remeš et al.: Adaptation and constraint shape the evolution of growth strategies in passerine birds across the globe.

## Data Availability

All data generated or analyzed during this study are included in this published article [and its supplementary information files].
