# Supplementary material for: Adaptation and constraint shape the evolution of growth patterns in passerine birds across the globe
Source: Front Zool. 2020 Sep 30;17:29. doi: 10.1186/s12983-020-00377-7 (PMC7526225; doi:10.1186/s12983-020-00377-7)
Supplement: Supplementary file 2 — Additional file 2: Figure S1. Sigmoid growth curves used to quantify peak growth rates. Figure S2. Asymptotes vs. adult trait values (Logistic). Figure S3. Asymptotes vs. adult trait values (Richards). Figure S4. Growth trajectories of 50 passerines. Figure S5. Average growth rates estimated using two different methods. Figure S6. Repeatability of peak growth rates across populations. Figure S7. Repeatability of fledging trait values across populations. Figure S8. Distribution of study populations across the world. Figure S9. Relative development of traits at fledging across species of passerines. Figure S10. Relative development of traits at fledging in relation to adult trait values in passerines. Figure S11. Fledging mass and fledging tarsus, wing, and tail length in relation to adult trait values in passerines. Figure S12. Relative development of traits at fledging in relation to fledging age in passerines. Figure S13. Relative development of traits at fledging in relation to residual fledging age in passerines. Figure S14. Peak growth rates (parameter K of the U-Logistic function) vs. fledging age in passerines. Figure S15. Peak growth rates (parameter K of the U-Richards function) vs. fledging age in passerines. Figure S16. Residual peak growth rates (parameter K of the U-Logistic function) vs. residual fledging age in passerines. Figure S17. Residual peak growth rates (parameter K of the U-Richards function) vs. residual fledging age in passerines. Figure S18. Relative development of traits at fledging in relation to residual peak growth rate (parameter K of the U-Logistic function). Figure S19. Relative development of traits at fledging in relation to residual peak growth rate (parameter K of the U-Richards function). [file 12983_2020_377_MOESM2_ESM.docx]

**Additional File 2 – Supplementary Figures**

**TITLE** Adaptation and constraint shape the evolution of growth strategies in passerine birds across the globe

**AUTHORS** Vladimír Remeš, Beata Matysioková and Jakub Vrána

**LIST OF FIGURES**

**Figure S1** Sigmoid growth curves used to quantify peak growth rates.

**Figure S2** Asymptotes vs. adult trait values (Logistic).

**Figure S3** Asymptotes vs. adult trait values (Richards).

**Figure S4** Growth trajectories of 50 passerines.

**Figure S5** Average growth rates estimated using two different methods.

**Figure S6** Repeatability of peak growth rates across populations.

**Figure S7** Repeatability of fledging trait values across populations.

**Figure S8** Distribution of study populations across the world.

**Figure S9** Relative development of traits at fledging across species of passerines.

**Figure S10** Relative development of traits at fledging in relation to adult trait values in passerines.

**Figure S11** Fledging mass and fledging tarsus, wing, and tail length in relation to adult trait values in passerines.

**Figure S12** Relative development of traits at fledging in relation to fledging age in passerines.

**Figure S13** Relative development of traits at fledging in relation to residual fledging age in passerines.

**Figure S14** Peak growth rates (parameter *K* of the U-Logistic function) vs. fledging age in passerines.

**Figure S15** Peak growth rates (parameter *K* of the U-Richards function) vs. fledging age in passerines.

**Figure S16** Residual peak growth rates (parameter *K* of the U-Logistic function) vs. residual fledging age in passerines.

**Figure S17** Residual peak growth rates (parameter *K* of the U-Richards function) vs. residual fledging age in passerines.

**Figure S18** Relative development of traits at fledging in relation to residual peak growth rate (parameter *K* of the U-Logistic function).

**Figure S19** Relative development of traits at fledging in relation to residual peak growth rate (parameter *K* of the U-Richards function).

**Figure S1.** Comparison of the cumulative increase and the relative growth rate of the three-parameter sigmoid growth curves (special cases of the U-Richards) having identical trait values at hatching = 0.1 (at age = 0), upper asymptotes *A* = 1, and peak growth rates at inflection *K* = 0.1. The comparison shows how the time of inflection (maximum relative growth, *t_i_*) differs between the models.

**Figure S2.** Correlations between the **asymptote** *A* of the Logistic growth model and **adult trait values**. “Body mass (70%)” means that body mass in the nest was truncated at 70% of adult body mass when fitting the sigmoid growth function (see Methods). Sample sizes are as follows: body mass 230 species, body mass (70%) 191 species, tarsus length 166 species, and wing length 128.

**Figure S3.** Correlations between the **asymptote** *A* of the Richards growth model and **adult trait values**. “Body mass (70%)” means that body mass in the nest was truncated at 70% of adult body mass when fitting the sigmoid growth function (see Methods). Sample sizes are as follows: body mass 201 species, body mass (70%) 122 species, tarsus length 147 species, and wing length 111.


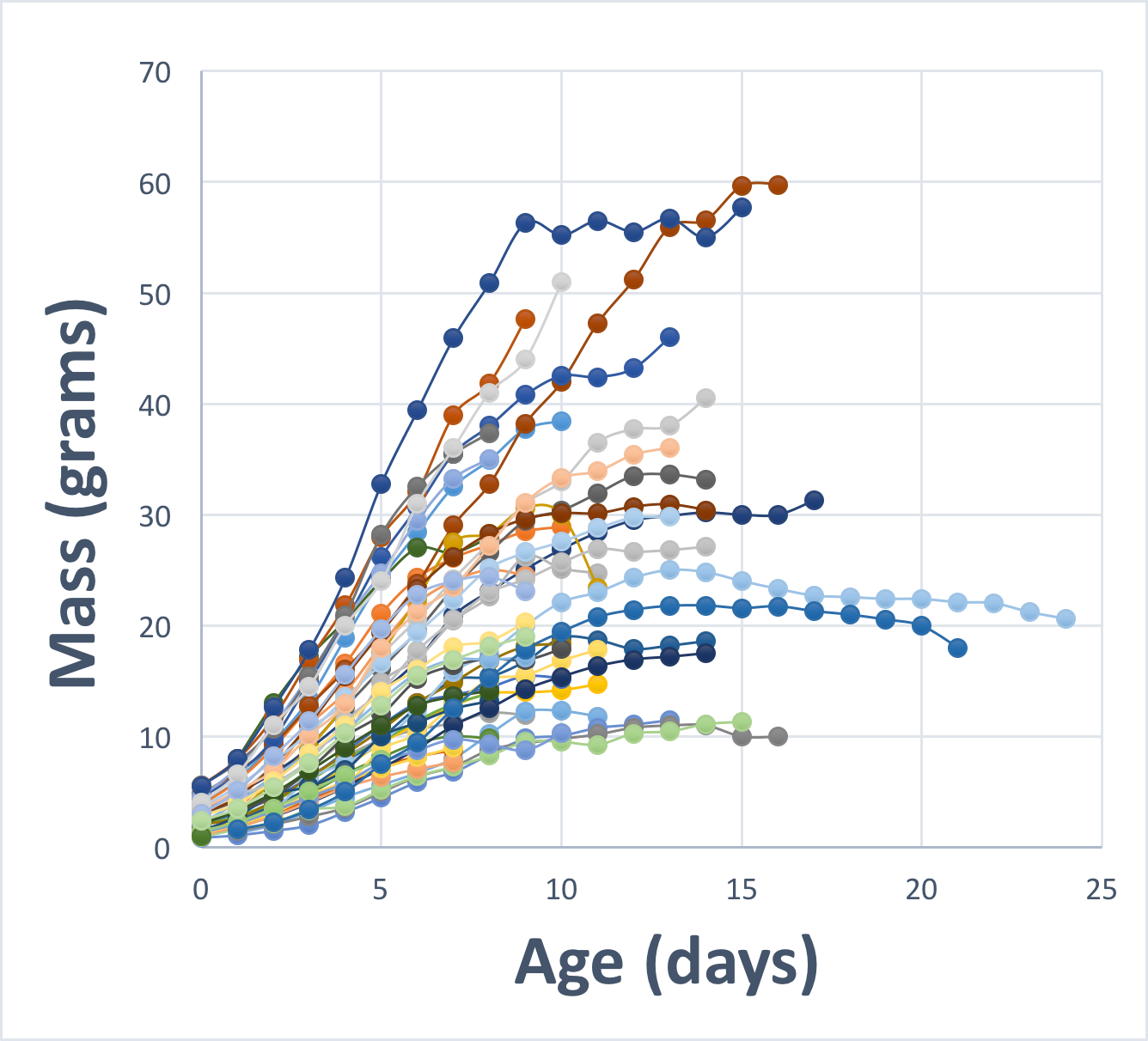

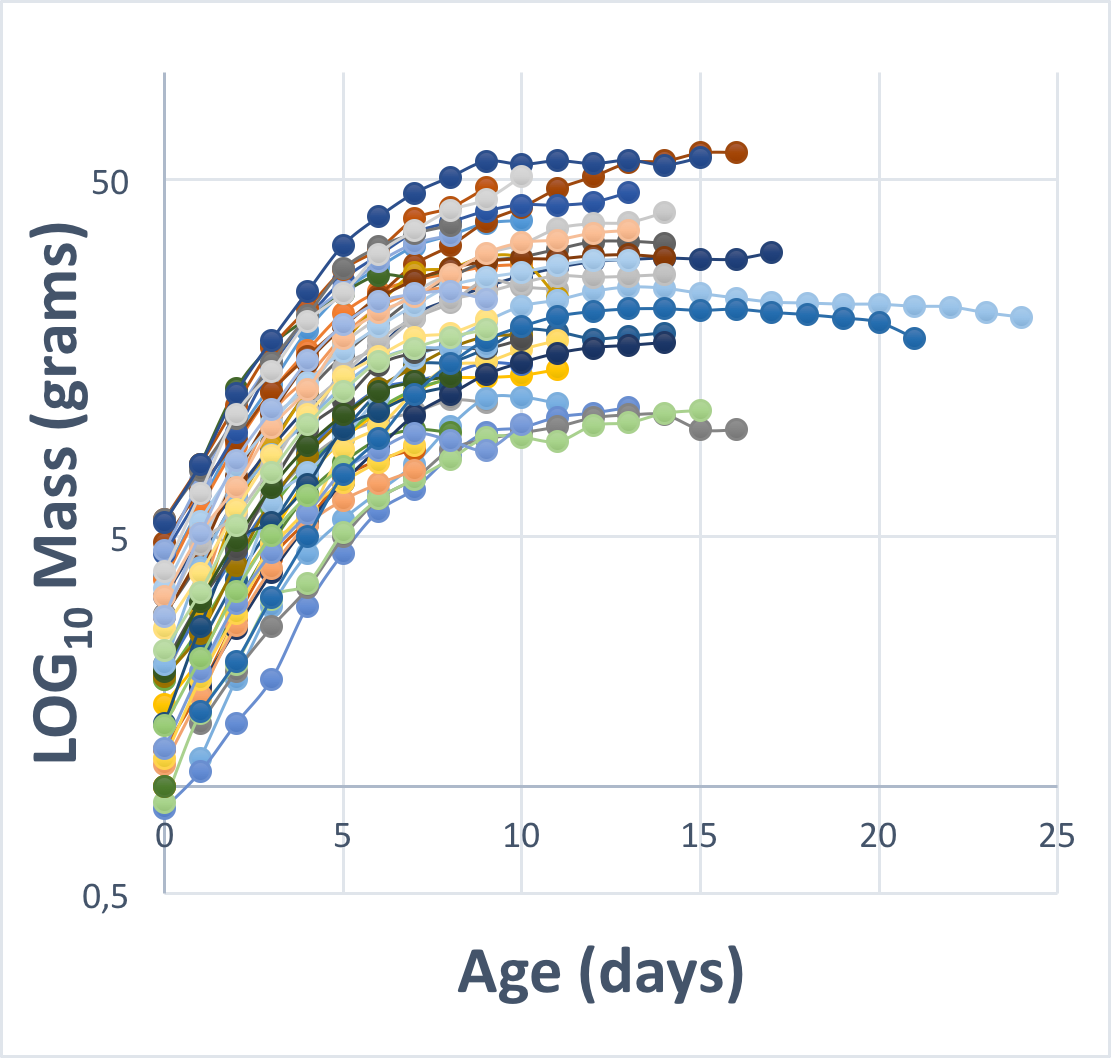


**Figure S4.** A sample of 50 **growth curves** of North American passerines with different growth rates and asymptotic masses representing typical growth patterns encountered in passerines in general. It shows that growth trajectory is typically sigmoid on linear scale (left) while decelerating on the log-scale (right). Thus, relative growth rate (in g g^-1^ day^-1^) is not constant over the time in the nest, but decreases (i.e., the derivative on the log-scale decreases with time in the nest).

**Figure S5.** Taking into account **hatching mass**: the correlation of **average growth rates** of body mass estimated as log10(fledging mass)/fledging time (“unadjusted”) vs. those estimated as log10(fledging mass/hatching mass)/fledging time (“adjusted”). The unadjusted growth rates are elevated compared to adjusted, but the correlation coefficient between the two is high (r = 0.81, n = 200 species), justifying the use of unadjusted estimates in main analyses.

**Figure S6.** **Repeatability** of **peak growth rates** (parameter *K*) across populations calculated as intraclass correlation coefficients (ICC). “All pops” are all populations available for a given species, while “Pops with n_pull>4” are populations with a minimum of 5 nestlings measured. Large black dots with thick black line are grand means of individual repeatabilities.

**Figure S7.** **Repeatability** of **fledging trait values** across populations calculated as intraclass correlation coefficients (ICC). “All pops” are all populations available for a given species, while “Pops with n_pull>4” are populations with a minimum of 5 nestlings measured. Large black dots with thick black line are grand means of individual repeatabilities.

**Figure S8.** **Distribution of study populations across the world**. RED color denotes populations with at least five measured nestlings (n = 295) and these were used in all analyses. ORANGE color denotes populations with less than five measured nestlings (n = 77). YELLOW color denotes populations with an unknown number of measured nestlings (n = 84). Both orange and yellow populations were excluded from analyses.

**Figure S9.**  **Relative development of traits at fledging** across species of passerines. The value of 1 means that the trait was developed at 100% of adult value, and this is designated by a vertical red line. Sample sizes are as follows: body mass 231 species, tarsus length 170 species, wing length 129 species, and tail length 72 species.

**Figure S10.**  **Relative development of traits at fledging** in relation to **adult trait values** in passerines. Sample sizes are as follows: body mass 231 species, tarsus length 170 species, wing length 129 species, and tail length 72 species. The value of 1 means that the trait was developed at 100% of adult value, and this is designated by a horizontal red line. Mass is in grams, while lengths are in mm.

**Figure S11.**  **Fledging mass** (grams) and **fledging tarsus, wing, and tail length** (mm) in relation to **adult trait values** in passerines. Sample sizes are as follows: body mass 231 species, tarsus length 170 species, wing length 129 species, and tail length 72 species.

**Figure S12.**  **Relative development of traits at fledging** in relation to **fledging age** in passerines. Sample sizes are as follows: body mass 231 species, tarsus length 170 species, wing length 129 species, and tail length 72 species. The value of 1 means that the trait was developed at 100% of adult value, and this is designated by a horizontal red line.

**Figure S13.**  **Relative development of traits at fledging** in relation to **residual fledging age** in passerines. Fledging age is residuals from PGLS regression of fledging age on adult body mass. Sample sizes are in Table S4. The value of 1 means that the trait was developed at 100% of adult value, and this is designated by a horizontal red line.

**Figure S14**. **Peak growth rates** (parameter *K* of the U-Logistic function) vs. **fledging age** in passerines. Sample sizes are as follows: body mass 230 species, body mass (70%) 191 species, tarsus length 174 species, and wing length 131 species. “Mass growth rate (70%)” means that body mass in the nest was truncated at 70% of adult body mass when estimating the peak growth rate (see Methods).

**Figure S15.**  **Peak growth rates** (parameter *K* of the U-Richards function) vs. **fledging age** in passerines. Sample sizes are as follows: body mass 201 species, body mass (70%) 122 species, tarsus length 151 species, and wing length 113 species. “Mass growth rate (70%)” means that body mass in the nest was truncated at 70% of adult body mass when estimating the peak growth rate (see Methods).

**Figure S16.**  **Residual peak growth rates** (parameter *K* of the U-Logistic function) vs. **residual fledging age** in passerines. Sample sizes are in Table S5. “Body mass” means that body mass in the nest was truncated at 70% of adult body mass when estimating the peak growth rate (see Methods). Growth rates are residuals from PGLS regressions of growth rate on adult value of a give trait, while fledging age is residuals from PGLS regression of fledging age on adult body mass.

**Figure S17.**  **Residual peak growth rates** (parameter *K* of the U-Richards function) vs. **residual fledging age** in passerines. Sample sizes are in Table S5. “Body mass” means that body mass in the nest was truncated at 70% of adult body mass when estimating the peak growth rate (see Methods). Growth rates are residuals from PGLS regressions of growth rate on adult value of a give trait, while fledging age is residuals from PGLS regression of fledging age on adult body mass.

**Figure S18.**  **Relative development of traits at fledging** in relation to **residual peak growth rate** (parameter *K* of the U-Logistic function). Growth rates are residuals from PGLS regressions of growth rate on adult value of a give trait. Sample sizes are in Table S6. “Body mass (70%)” means that body mass in the nest was truncated at 70% of adult body mass when estimating the peak growth rate (see Methods). The value of 1 means that the trait was developed at 100% of adult value, and this is designated by a horizontal red line.

**Figure S19.**  **Relative development of traits at fledging** in relation to **residual peak growth rate** (parameter *K* of the U-Richards function). Growth rates are residuals from PGLS regressions of growth rate on adult value of a given trait. Sample sizes are in Table S6. “Body mass (70%)” means that body mass in the nest was truncated at 70% of adult body mass when estimating the peak growth rate (see Methods). The value of 1 means that the trait was developed at 100% of adult value, and this is designated by a horizontal red line.
